# Naringin ameliorates motor dysfunction and exerts neuroprotective role against vanadium-induced neurotoxicity

**DOI:** 10.3934/Neuroscience.2022031

**Published:** 2022-12-26

**Authors:** Adeshina O. Adekeye, Adedamola A. Fafure, Ayoola E. Ogunsemowo, Linus A. Enye, Olusola S. Saka, Oluwatosin O. Ogedengbe

**Affiliations:** 1 Department of Anatomy, College of Medicine and Health Sciences, Afe Babalola University, Ado-Ekiti, Ekiti State, Nigeria; 2 Department of Anatomy, Faculty of Basic Medical Sciences, Federal University Oye-Ekiti, Ekiti State, Nigeria

**Keywords:** substantia nigra, caudate putamen, Parkinson's disease, vanadium, Naringin

## Abstract

Exposure to vanadium has been known to lead to a progressive neurodegenerative disorder like Parkinson's disease. Naringin is a known flavonoid glycoside that is mostly seen in the flesh of grapefruit and orange and is believed to have protective effects for the treatment of neurodegenerative disorders. This study sought to investigate the role of Naringin in the treatment of vanadium-induced neurotoxicity. Vanadium (10 mg/kg BW) was injected intraperitoneally to induce motor dysfunction, followed by treatment with Naringin (30 mg/kg BW) intraperitoneally for 14 days. Oxidative stress imbalance was monitored by checking Glutathione Peroxidase (GPX) and Catalase levels. Histological and immunohistochemical alterations were observed using RBFOX3 polyclonal antibody to determine neuronal cell distribution and NLRP3 inflammasome antibody as a marker of inflammation. Exposure to vanadium induces neurotoxicity by significantly increasing the Catalase and Glutathione Peroxidase (GPX) levels. Vanadium administration also led to increased inflammatory cells and a significant reduction of the viable neuronal cells in the SNc and CPu. Treatment with Naringin showed a neuroprotective role by dependently restoring the Catalase and Glutathione Peroxidase (GPX) levels, inflammasome activation, and neuronal damage in the SNc and CPu. Naringin demonstrated anti-oxidative, and anti-inflammatory responses by inhibiting oxidative stress, and inflammation and exerts neuroprotective effects by inhibiting apoptosis following vanadium-induced neurotoxicity in adult Wistar rats.

## 1. Introduction

Vanadium is a trace element that has a function in living organisms most especially plants and animals, by acting as a growth promoting factor in young animals and stimulating chlorophyll synthesis in plants [1]. The role of vanadium in biological systems depends on the biophysical state especially the valence state and solubility, the dose, route and duration of exposure [2]. Vanadium can be released into the atmosphere at high amounts through man made sources like burning of fossil fuels, spillage of heavy oils especially in places like Niger delta Nigeria, one of the largest petroleum rich region in Africa [3]. One of the major public health hazards that has been associated with neurological and oxidative damage is dietary and occupational exposure of vanadium [6]. The dietary exposure of vanadium can be as a result of consuming infected food and water, while occupational exposure can be through air, or typical factory system. It has been reported that vanadium is needed in lower dose in the body for normal bone growth, and it may significantly lower blood sugar levels in patient with type 2 diabetes, it has been used as a treatment of heart disease, tuberculosis, cancer, syphilis, and has been reported to improved athletic performance in weight training. However, exposure to higher dose of vanadium due to increasing mining, smelting, weathering of rocks, sediments rich in vanadium, and infected food may pose a major health concern [2]. This higher concentration might enter the body system through the lungs and stomach, where it's been taking-up via the blood stream and been deposited in the brain. Its accumulation in the brain might induces oxidative stress and has a result, promote apoptotic cell death in the substantia nigral dopaminergic neurons, which leads to early pathogenesis of Parkinson's disease [1]. Naringin (NAR) is a well-known flavanone glycoside of tropical and subtropical fruits. It has different biological and pharmacological influence such as anti-inflammatory, antioxidant and lipid-lowering activities. It is also known to possess neuroprotective effect against neurodegenerative disorders through modulating the oxidative stress and inflammatory responses [4],[5]. Studies on NAR against neurodegenerative disease showed its anti-apoptoticproperty by inducing the glucose regulated protein 78 (GRP78), the endoplasmic reticulum (ER) chaperone and anti-inflammatory property by reducing the inflammatory cytokines and free radicals. All these findings pointed out that NAR can be considered as potential neuroprotective agent through its antioxidant activity [6]. This study aim to determine anti-inflammatory and anit-oxidative response of Naringin, the neuronal cells distribution pattern in the Substantia nigra (SNc) and Caudate Putamen (CPu) following vanadium induced neurotoxicity.

## 2. Materials and methods

### 2.1. Groups and treatments

A total of 40 healthy male Wistar rats (age: 18 weeks, weight: 150–170 g), gotten from animal holdings of the Department of Anatomy, Afe Babalola University, Ado-Ekiti, were used for this research. They were placed in a standard laboratory rat cage under normal light and dark circle with access to pellet-fed with water ad libitum. All procedures were precisely in agreement with the guidance in the Guide for the Care and Use of Laboratory Animals of the National Institutes of Health, and the Department of Human Anatomy, College of Medical and Health Sciences, Afe Babalola University Ado Ekiti, Nigeria (AB/EC/22/01/98).

The rats were randomly divided into four groups (n = 10). Group A (Control: normal saline), Group B (Naringin at 30 mg/kg BW intraperitoneally), Group C (Naringin at 30 mg/kg BW and Vanadium at 10mg/kg BW), Group D (Vanadium at 10mg/kg BW intraperitoneally). The duration of the experiment was 14 days [7],[8].

### 2.2. Chemicals and Reagents

RBFOX3 and NLRP3 polyclonal antibody (Elabscience), 2-step plus poly-HRP anti-mouse/rabbit IgG Detection system (Elabscience), DAB solution, Triton-X, phosphate buffered formal saline (Elabscience), Hematoxylin and eosin (Bisolab), Ethanol and Xylene (Bisolab).

### 2.3. Animal sacrifice and sample preparation

After the last administration, the animals were anesthetized by intraperitoneal injection of 10 mg/kg BW ketamine hydrochloride. The animals were sacrificed through intra-cardiac perfusion fixation. The brain was removed, and fixed inside 4% paraformaldehyde, the region of Substantia nigra and caudate putamen was grossed and post-fixed, and stored at room temperature until needed for histological and immunohistochemical analysis. The brain sections for biochemical analysis was homogenized and centrifuged at 10,000 rpm for 10 min at 4 °C. The supernatant were isolated and used to access Glutathione Peroxidase and Catalase activities.

### 2.4. Histological and Immunohistochemical Analysis

The experimental animals were sacrificed 24 hours after the last administration under ketamine anesthesia. For immunohistochemistry studies sections of the caudate putamen and the substantia nigra (Section: 5 µm thick) were coronally section with a rotary microtome. Endogenous peroxidase activity within the tissue sections were briefly removed by incubating the sections in 3% H_2_O_2_ for 10 min at 37 °C after regular dewaxing and dehydration. According to the Elabscience protocol, sections were immune-stained with primary antibodies directed againstneuronal cells (RBFOX3 polyclonal antibodies) to determine neuronal cell distribution, and inflammation (NLRP3 polyclonal antibodies) to mark for inflammatory cells (NLRP3 1:200; RBFOX3 1:150, Elabscience, china).

### 2.5. Microscopy Analysis

An OPTO-Edu industrial camera light microscope connected to a computer with an image-processing and analysis software Image-J (Version 1.53) were used to analyze the photomicrograph. The image were enhanced using oil immersion and the system was adjusted to obtain clarity and resolution. In a given square area, the total positive cells was counted and calculated. 30 sections (5 µm) of the SNc and CPu in each animal were analysed. With the aid of the software mentioned above, a non-destructive grid of lines was set to guide against counting the same cells twice.

### 2.6. Statistical Analysis

Differences among multiple groups were statistically analyzed using One-way ANOVA and post hoc Turkey test. Graph Pad Prism 5 (Version 5.03, Graphpad Inc.) was the statistical package used for data analysis. Significant difference was set at p < 0.05.

## 3. Results

### 3.1. Biochemical Analysis

Oxidative stress markers, glutathione peroxidase (GPX) and Catalase, were evaluated in the brain of rat following the administration of vanadium to induce neurotoxicity.

### 3.2. GPX

The results of the analysis show that exposure to VAN decreased glutathione peroxidase levels as seen in VAN group and treatment with Naringin was effective in improving GPX levels as seen in NAR+VAN group.

**Figure 1.**
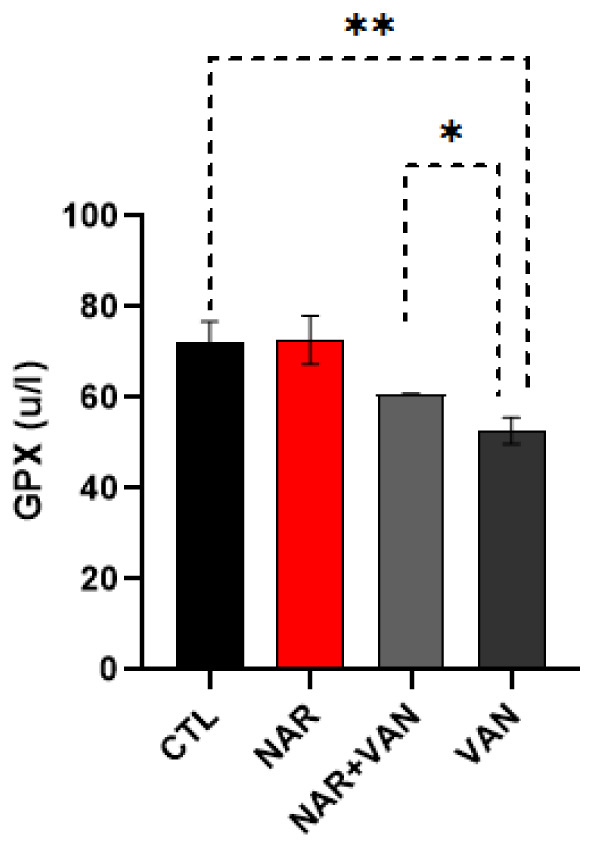
Bar chart representation showing changes in the brain GPX level following exposure to VAN and treatment with naringin. Comparison between groups by one-way ANOVA followed by Tukey's multiple comparison test shows a decrease in the brain glutathione peroxidase level in VAN group when compared with CTL group (**p < 0.01). Treatment with Naringin shows a significant increase in the brain glutathione levels in NAR+VAN group when compared to VAN group (*p < 0.05). Legend: CTL = control group; NAR = naringin group; NAR + VAN = Naringin+Vanadium group; VAN = vanadium group.

### 3.3. Catalase

The results of the analysis show that exposure to VAN decreased catalase levels as seen in VAN group and treatment with Naringin was effective in improving catalase levels as seen in NAR+VAN group.

**Figure 2.**
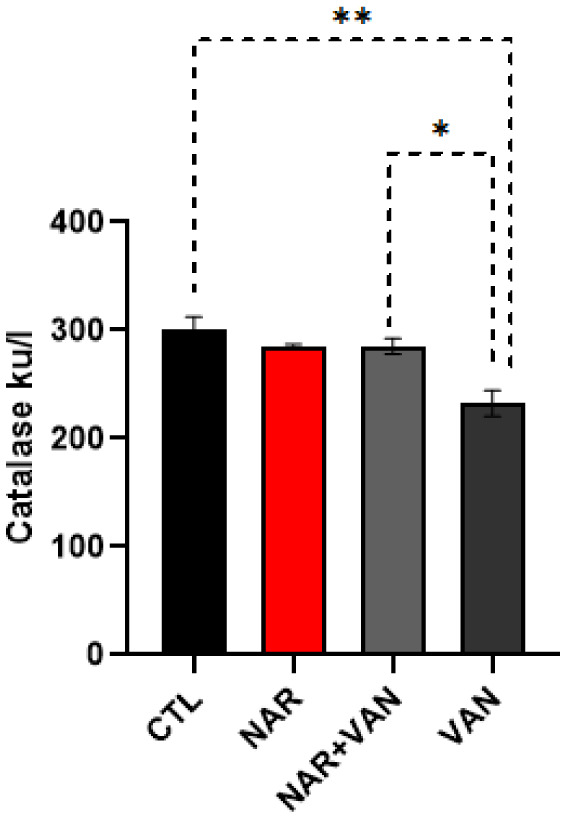
Bar chart representation showing changes in the brain catalase level following exposure to VAN and treatment with naringin. Comparison between groups by one-way ANOVA followed by Tukey's multiple comparison test shows a significant decrease in the brain catalase level in VAN group when compared with CTL group (**p < 0.01). Treatment with Naringin increased the brain catalase levels in NAR+VAN group when compared to VAN group. There is equally a significant increase in the brain catalase level in rat exposed to just Naringin in the NAR group when compared to VAN group (*p < 0.05). Legend: CTL = control group; NAR = naringin group; NAR + VAN = Naringin + Vanadium group; VAN = vanadium group.

### 3.4. Histological Analysis

Figure 3a revealed the efficacy of naringin in the substantia nigra of vanadium treated animals. Control and naringin group revealed a normal distribution of neuronal cell and a statistically significant increase in the cell count when compared to the vanadium group, which shows a reduction in the cell count. Naringin treated group demonstrated a statistically significant increase in the cell count when compared to the vanadium group. The slide below demonstrated the role of naringin in the caudate putamen of vanadium treated animals. Control and naringin group revealed a normal distribution of neuronal cell and a statistically significant increase in the cell count when compared to the vanadium group, which shows a reduction in the cell count. Naringin treated group demonstrated a statistically significant increase in the cell count when compared to the vanadium group (Figure 3b).

**Figure 3a.**
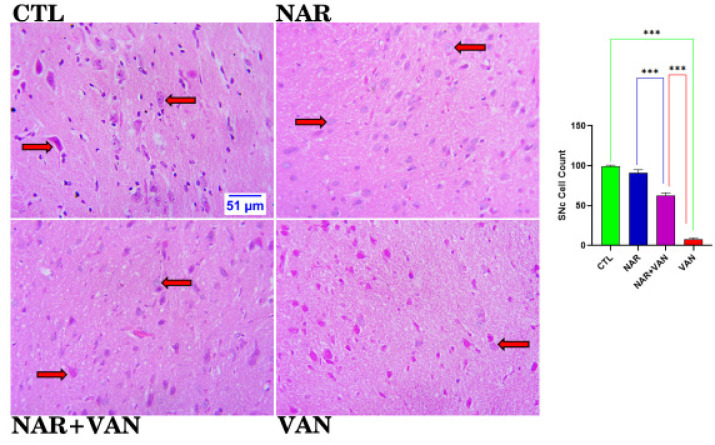
Histoarchitectural changes and cell count of the Substantia Nigra (SNc) showing the neuronal cell morphology and distribution. CTL and NAR group revealed a normal distribution of neuronal cell as well as a statistically significant increase in the cell count when compared to vanadium treated group. VAN group showed neuronal cells undergoing apoptosis, and a statistically significant reduction in the count when compared to CTL. However, treatment with naringin was able to ameliorate the negative impact of vanadium. H&E. (Mag. x800; Scale bar: 51 µm), (***p < 0.001). Legend: CTL = control group; NAR = naringin group; NAR + VAN = Naringin + Vanadium group; VAN = vanadium group.

**Figure 3b.**
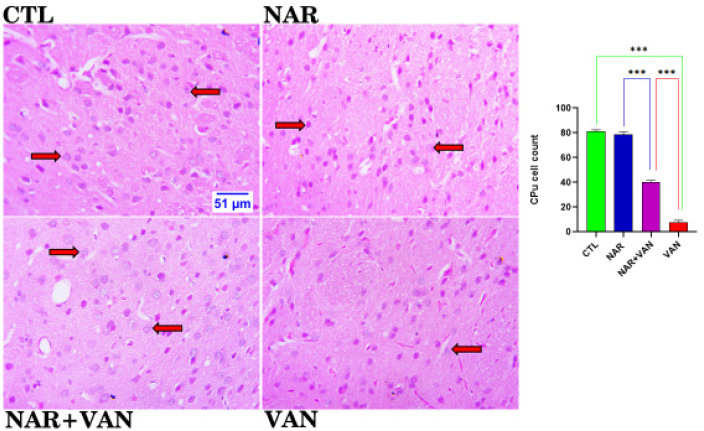
Histoarchitectural changes and cell count of the Caudate Putamen (CPu) showing the neuronal cell morphology and distribution. CTL and NAR group revealed a normal distribution of neuronal cell as well as a statistically significant increase in the cell count when compared to vanadium treated group. VAN group showed neuronal cells undergoing apoptosis, and a statistically significant reduction in the count when compared to CTL. However, treatment with naringin was able to ameliorate the negative impact of vanadium. H&E. (Mag. x800; Scale bar: 51 µm), (***p < 0.001). Legend: CTL = control group; NAR = naringin group; NAR + VAN = Naringin + Vanadium group; VAN = vanadium group.

### 3.5. Immunohistochemistry

Immunohistochemical study on caudate putamen and substantia nigra evaluating the positive impact of naringin following vanadium neurotoxicity.

### 3.6. NeuN for Substantia Nigra (SNc)

**Figure 4a.**
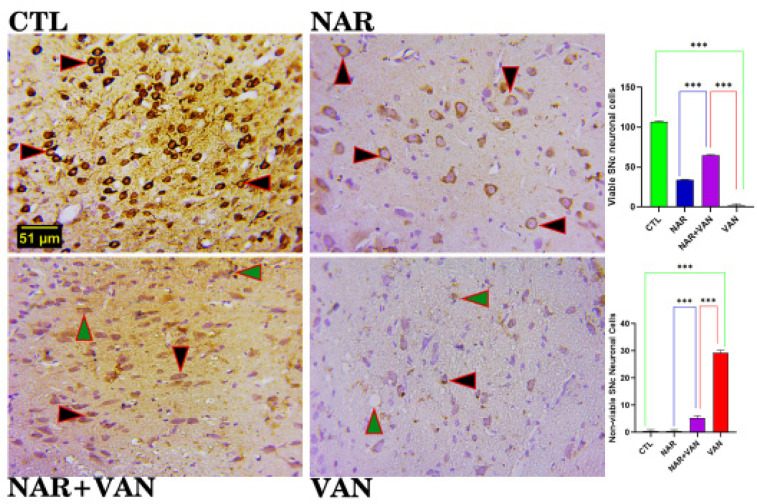
NeuN viable cells in the Substantia Nigra (SNc). CTL and NAR group revealed a lot of viable nerve cells as shown in the photomicrograph. In the NAR+VAN group and the VAN group very little amount of viable cells are visible. (Dark brownish round cells indicate the positive nerve cells). Bar chart representation showing the cell count of viable cells following exposure to VAN and treatment with naringin. Comparison between groups by one-way ANOVA followed by Tukey's multiple comparison test shows a significant decrease in viable cells in VAN group when compared with CTL group (***p < 0.001). Treatment with Naringin increased the immunopositive cell count in NAR+VAN group when compared to VAN group (***p < 0.001). There is equally an increase in the immunopositive cell count in rat exposed to just Naringin in the NAR group when compared to VAN group (***p < 0.001). Legend: CTL = control group; NAR = naringin group; NAR + VAN = Naringin + Vanadium group; VAN = vanadium group. (Mag. x800; Scale bar: 51 µm)

### 3.7. NeuN for Caudate Putamen (CPu)

**Figure 4b.**
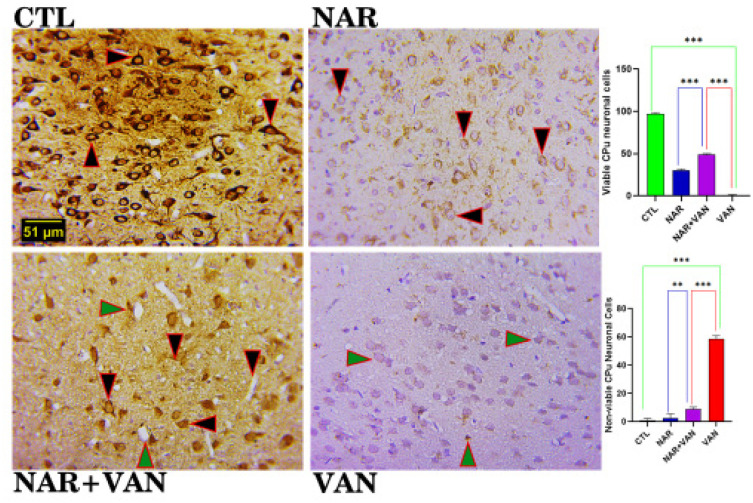
Neun viable cells and Non-Viable cells in the Caudate Putamen (CPu). CTL and NAR group revealed many viable nerve cells as shown in the photomicrograph. In the NAR+VAN group and the VAN group very little amount of viable cells were seen. (Dark brownish round cells indicate viable nerve cells). Bar chart representation showing the cell count of viable cells following exposure to VAN and treatment with naringin. Comparison between groups by one-way ANOVA followed by Tukey's multiple comparison test shows a significant decrease in viable cells in VAN group when compared with CTL group (***p < 0.001). Treatment with Naringin increased the immunopositive cell count in NAR+VAN group when compared to VAN group (***p < 0.001). There is equally an increase in the immunopositive cell count in rat exposed to just Naringin in the NAR group when compared to VAN group (***p < 0.001). Legend: CTL = control group; NAR = naringin group; NAR + VAN = Naringin + Vanadium group; VAN = vanadium group. (Mag. x800; Scale bar: 51 µm)

### 3.8. NLRP3 Inflammasome for SNc

**Figure 5a.**
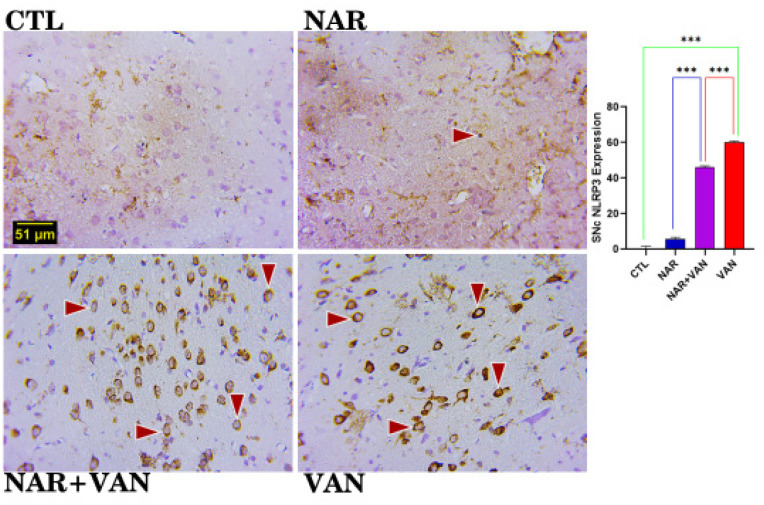
Inflammasome immunopositive cells in the Substantia Nigra (SNc). CTL and NAR group revealed less or no expression of inflammasome as shown in the photomicrograph. Expression of inflammasome cells are visible in the NAR+VAN group and the VAN group. (Dark brownish round cells indicate the immunopositive cells). Bar chart representation showing the cell count following exposure to VAN and treatment with naringin. Comparison between groups by one-way ANOVA followed by Tukey's multiple comparison test shows a significant increase in inflammasome in VAN group when compared with CTL group (***p < 0.001). Treatment with Naringin reduced the immunopositive cell count in NAR+VAN group when compared to VAN group (***p < 0.001). There is equally a significant reduction in the immunopositive cell count in rat exposed to just Naringin in the NAR group when compared to VAN group (***p < 0.001). Legend: CTL = control group; NAR = naringin group; NAR + VAN = Naringin + Vanadium group; VAN = vanadium group. (Mag. x800; Scale bar: 51 µm)

### 3.9. NLRP3 Inflammasome for Caudate Putamen (CPu)

**Figure 5b.**
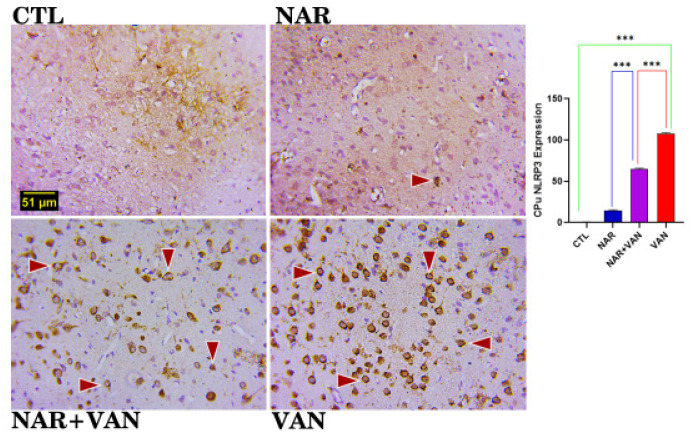
Inflammasome immunopositive cells in the Caudate Putamen (CPu). CTL and NAR group revealed less or no expression of inflammasome as shown in the photomicrograph. Expression of inflammasome cells are visible in the NAR+VAN group and the VAN group. (Dark brownish round cells indicate the immunopositive cells). Bar chart representation showing the cell count following exposure to VAN and treatment with naringin. Comparison between groups by one-way ANOVA followed by Tukey's multiple comparison test shows a significant increase in inflammasome in VAN group when compared with CTL group (***p < 0.001). Treatment with Naringin reduced the immunopositive cell count in NAR+VAN group when compared to VAN group (***p < 0.001). There is equally a significant reduction in the immunopositive cell count in rat exposed to just Naringin in the NAR group when compared to VAN group (***p < 0.001). Legend: CTL = control group; NAR = naringin group; NAR + VAN = Naringin + Vanadium group; VAN = vanadium group. (Mag. x800; Scale bar: 51 µm).

## 4. Discussion

In this research, the motor benefit of Naringin in a mouse model of vanadium-induced neurotoxicity which is similar to parkinsonism is evaluated. Oxidative stress is one of the major factors contributing to Parkinson's disease, Naringin is beneficial because it is believed to be dopamine enhancing and a potent antioxidant. The data provided suggest that Naringin could have a beneficial effect in the treatment of motor deficit induced by vanadium.

Oxidative stress is known to have an effect in neurodegenerative disorders like Parkinson's disease where damage to neurons can reflect both an increase in oxidative processes and a decrease in antioxidant defenses. Oxidative stress is defined as the imbalance between biochemical processes leading to the production of reactive oxygen species [9]. The ROS species like superoxide, hydrogen peroxide, and hydroxyl radical ions, oxidize the bio-chemicals and destroy lipids, DNA and RNA, which can then cause neurodegeneration [10]. In this study the effect of Naringin on the Catalase and Glutathione peroxidase (GPx) levels in the Substantia nigra and Caudate Putamen of rat brain after the exposure to vanadium was evaluated. GPx is one of the most abundant antioxidants that can be found in the brain, it is essential in the whole defense approach of antioxidants, more importantly as regards to superoxide anion radical (*O_2_) which is generated through mitochondrial energy synthesis pathway [22]. In the result, GPX levels significantly reduced when comparing the control group with Vanadium only group as seen in figure 1 (*P < 0.05). This result agrees with Adekeye et al. [8] that vanadium decreases GPx levels, which might be as a result of increase in the formation of free radicals which then causes oxidative stress. Following treatment with Naringin there was a significant increase in GPx level in the treated group NAR+VAN when compared to the neurotoxic group (VAN). This result agrees with Garabadu and Agrawal, [11] on the ability of Naringin to improve GPx levels in Parkinson's induced rat by activating the nuclear factor erythroid 2-related factor 2 (Nrf2) signaling pathway in neurons. Results from the catalase levels when comparing the control group to Parkinson's induced group (VAN) show a significant decreased level of catalase in the VAN group as seen in figure 2 (**P < 0.01). This result corresponds with Adebiyi et al. [12]. Treatment with Naringin brought about a statistically significant increase in the treated group NAR+VAN when compared to the VAN group. This is in accordance to sugumar et al. [13] as Naringin showed effectiveness in restoring catalase levels.

From the histological slides of Substantia nigra (SNc) and Caudate Putamen (CPu), normal neuronal cells with centrally located nuclei are visible in control (CTL) and Naringin (NAR) group, Figure 3a and b. VAN group showed neuronal cells undergoing apoptosis, and a statistically significant reduction in the count when compared to CTL group (***P < 0.001). This result agrees with Folarin et al. [14] who stipulated that vanadium was able to reduce the neuronal cells in the brain following cell count as a result of the up-regulation of glia cells and this allows for apoptosis of neurons. The NAR+VAN group revealed neuronal cells undergoing recovery with centrally located nuclei and an increase in the count when compared to VAN group. This result agrees with Uddin et al. [15] that Naringin can act as an anti-apoptotic drug in neurodegeneration by inducing Glial cell line-derived neurotrophic factor (GDNF) which is a protein that allows for the survival of nerve cells that have been lost as a result of the induced neurotoxicity. It is evident from this study that vanadium causes degenerative changes in the Substantia nigra (SNc) and Caudate putamen (CPu) which involves loss of neuronal cells with impaired motor function, and this is in line with statement made in Nnama et al. [16].

In Immunohistochemistry, NeuN and NLRP3 Inflammasome markers were used. Neuronal nuclear protein (NeuN) is expressed in the nuclei of majority of the nervous system neurons. It is regarded as an immunohistochemical marker commonly used for neurons. NeuN marks for viable and non-viable neurons and a comparisons between the groups can be achieved. NeuN immunohistochemistry is widely used to test for loss of phenotypic marker expression and cell-type specificity of the lesions in PD models [17]. In NeuN result of the Substantia Nigra (SNc) and the Caudate Putamen (CPu) control (CTL) and Naringin (NAR) revealed a large amount of viable cells with centrally located nuclei Figure 4a and 4b. In the NAR+VAN group and the VAN group there is visible amount of non-viable cells with VAN group having a significant higher number of non-viable cell and least amount of viable cells when compared to the NAR+VAN group (***P < 0.001). This result agrees with Zou et al. [18] that naringin can reduce neurodegeneration and restore neuronal development.

NLRP3 inflammasome major role is to detect inflammation and immune system-related disorders, and also in the pathogenesis of several neurodegenerative diseases [19]. In this study it is used to check inflammation in the cells of the Substantia nigra (SNc) and Caudate Putamen (CPu). In the result of figure 5a and 5b, there is a significantly high amount of NLRP3 positive cells in the VAN group when compared to the CTL group (***P < 0.001). The result agrees with Im et al. [20] that vanadium increases the activation of NLRP3 in cells which might be as a result of Tumour Necrosis Factor alpha (TNF alpha) that is produced. In the Naringin treated group (NAR+VAN) there is a decreased number of NLRP3 positive cell in the SNc and CPu when compared to the Parkinson's induced group (VAN), this just shows that Naringin inhibits the activation of NLRP3 and production of Tumour Necrosis Factor alpha [21].

## 5. Conclusion

The results from this study shows that vanadium was effective in altering motor function by lowering the antioxidant level, reducing neuronal cells and stimulating inflammation in adult Wistar rat, while, Naringin play an anti-oxidative and anti-inflammatory role by elevating the catalase and Glutathione peroxidase level, and reviving the neuronal cells and inflammatory cells.
